# Molecular characterisation of a novel sadwavirus infecting cattleya orchids in Australia

**DOI:** 10.1007/s00705-024-05980-1

**Published:** 2024-03-07

**Authors:** Nga T. Tran, Paul R. Campbell, Kathleen S. Crew, Andrew D. W. Geering

**Affiliations:** 1 Centre for Horticultural Science, Queensland Alliance for Agriculture and Food Innovation, The University of Queensland, Ecosciences Precinct, GPO Box 267, Brisbane, QLD 4001 Australia; 2 Department of Agriculture and Fisheries, Ecosciences Precinct, GPO Box 267, Brisbane, QLD 4001 Australia

## Abstract

The complete genome sequence of a novel sadwavirus infecting cattleya orchids in South East Queensland is described. Isometric virions of *c*. 27 nm diameter were observed in sap extracts viewed under a transmission electron microscope, and the genome sequence of this virus was determined by high-throughput sequencing. The viral genome consists of two RNA components, 5,910 and 4,435 nucleotides (nt) in length, each encoding a long polyprotein, with predicted cleavage sites at H/Y, E/G, Q/S, and Q/G for the RNA1 and T/G for the RNA2 translation products, respectively. RNA2 has an additional small ORF of 684 nt near the 3ʹ untranslated region. Phylogenetic analysis based on an amino acid sequence alignment of the Pro-Pol region suggested that this virus is most closely related to pineapple secovirus A, a member of the subgenus *Cholivirus*, but warrants classification as a member of a new species because it exhibited no more than 64% amino acid identity in pairwise sequence comparisons. Because of the prominent purple ringspots that were observed on the leaves of some of the plants, we propose the name “cattleya purple ringspot virus” for this virus (suggested species name: “*Sadwavirus cattleyacola*”).

**Supplementary Information:**

The online version contains supplementary material available at 10.1007/s00705-024-05980-1.

The family Orchidaceae is one of the two largest vascular plant families in the world, represented on all continents except Antarctica [3]. Many orchid species are cultivated by hobbyists and by commercial orchid nurseries, but many are still harvested illegally from the wild, driving some species to extinction [11]. It is estimated that about 10% of the international fresh flower trade consists of either potted orchid plants or freshly cut inflorescences [4]. Between 1996 and 2015, at least 1.1 billion live orchid plants and 31 million kg of flower stems were traded, with Taiwan and Thailand being the largest exporters, and conversely, South Korea, the USA, and Japan being the largest importers [8].

Orchids are susceptible to infection by a very large range of viruses, some of which are harmful pathogens that affect both the growth of the plant and the ornamental appeal of the flowers [14]. The most thoroughly researched of these viruses are cymbidium mosaic virus (CymMMV; genus *Potexvirus*), odontoglossum ringspot virus (ORSV; genus *Tobamovirus*), and orchid fleck virus (OFV; genus *Dichorhavirus*), all of which are found in Australia but mainly in cultivated orchids [1, 6, 7]. A distinct set of viruses infect wild orchid species in Australia [6], and most are considered native, such as caladenia virus A [12], ceratobium mosaic virus [7], donkey orchid symptomless virus [13], and pterostylis blotch virus [2].

In July 2021, diseased cattleya hybrid (‘Lumita’ × ‘Chia Lin Doll’) plants were received from an orchid grower in Gatton, Queensland. These plants had prominent, purple-pigmented ringspots and smaller spots and flecks on the leaves, indicative of a virus infection (Fig. 1a). The parental cross to create these hybrid plants was done by the grower, and the plants had been raised from true seed in aseptic culture and grown out in a shade-house. No disease symptoms were observed by the grower in either parent plant. Infection with CymMV or ORSV was ruled out by negative staining of a sap extract with 1% ammonium molybdate, pH 5.8, and examination under a JEOL JEM-1400 transmission electron microscope. However, both empty and full non-enveloped, isometric virions, *c*. 27 nm in diameter were observed (Fig. 1b). Symptomatic leaves from a diseased plant were freeze-dried and deposited in the Queensland Department of Agriculture and Fisheries (QDAF) Plant Virus Collection under isolate number 5854.

**Fig. 1:**
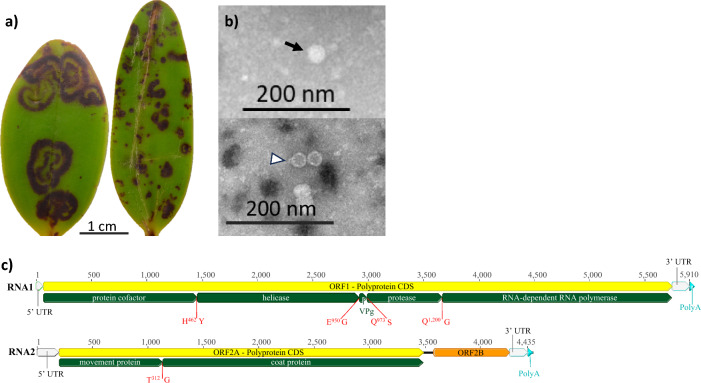
Characteristics of cattleya purple ringspot virus isolate 5854. (a) Symptoms observed on leaves of cattleya hybrid (‘Lumita’ × ‘Chia Lin Doll’) plants. (b) Isometric virions that were penetrated or not penetrated by the negative contrast solution are highlighted by an arrowhead or an arrow, respectively. (c) Genome maps of the RNA1 and RNA2 components showing open reading frames and putative protein domains with predicted amino acid cleavage sites shown in red. The scales on the genome maps represent the number of nucleotides

To identify the putative virus from isolate 5854, high-throughput sequencing was done. Total RNA was extracted using a TRIzol Plus RNA Purification Kit (catalog number 12183555, Invitrogen) as per the manufacturer’s instructions. The total RNA extract was submitted to the Australian Genome Research Facility (AGRF; Melbourne, Australia) for library preparation and sequencing. Ribosomal RNA was removed using a TruSeq Stranded Total RNA with Ribo‐Zero Plant Kit (Illumina) with 500 ng of input RNA. The library was sequenced as 150-bp paired-end reads on an Illumina MiSeq platform, using a 300-cycle kit. The total number of reads obtained was 1,301,670. The reads were paired, and adaptor and primer sequences were removed using the BBDuk Plugin for Geneious Prime v. 2021.0.1 (Biomatters Ltd., Auckland). Trimming parameters included removal of reads with a Phred value less than 30 and a length shorter than 30 nucleotides (nt). The remaining 1,021,594 reads were assembled *de novo* using CLC Genomics Workbench v. 6.5 (QIAGEN, USA) with automatic word size and bubble size and a minimum contig length of 700 nt.

After *de novo* sequence assembly, 724 sequence contigs were obtained, which were sorted by length and annotated with the closest matches from the NCBI Nucleotide Database following a BLASTn search. One contig, assembled from 3,501 reads and with an average sequence coverage depth of 79.2 reads, had significant similarity to the RNA1 sequence of pineapple secovirus A (GenBank accession MN809923) and chocolate lily virus A (GenBank accession JN052073). A second contig, assembled from 3,479 reads and with an average sequence coverage depth of 101.3 reads, had significant similarity to the RNA2 sequences of the same sadwaviruses. A maximum of 64% nt sequence identity was observed in partial sequence alignments (query coverage <13%) of the two contigs, suggesting that they were derived from a novel virus. No other sequence contigs had significant similarity to other viral sequences in GenBank.

To determine the 5ʹ and 3ʹ termini of the two RNA components, RACE was performed using virus-specific primers that were placed close to the presumed 5ʹ and 3ʹ ends of the sequences (Supplementary Table S1). Total RNA was extracted using a TRIzol Kit, residual DNA was eliminated by digestion with DNase I (catalog number M0303S, New England Biolabs), and a final purification done using a Monarch RNA Cleanup Kit (catalog number T2030L, New England Biolabs). Complementary DNA was prepared using Tag1 (underlined)-oligo(dT) primer (5ʹ CCACGCGTATCGATGTCGAC(T)_40_VN 3ʹ) and Maxima H Minus reverse transcriptase (catalog number EP0752, Thermal Fisher Scientific). The cDNA was then purified using a Monarch DNA Cleanup Kit (catalog number T1030L, New England Biolabs) and A-tailed, C-tailed, and G-tailed using terminal transferase (catalog number M0315S, New England Biolabs). The cDNA was purified a second time using a Monarch DNA Cleanup Kit, and 5ʹ and 3ʹ RACE were done using Q5 High Fidelity DNA Polymerase (catalog number M0494S, New England Biolabs) for the amplification reactions. For the first round of PCR for 5ʹ RACE, the Tag1oligo(dT), Tag1(G)_7_HN, or Tag1(C)_7_DN primer for A-tailed, C-tailed or G-tailed cDNA, respectively, was paired with a virus-specific reverse primer (Supplementary Table S1), and for the second round of PCR, the Tag1 primer (5ʹ CCACGCGTATCGATGTCGAC 3ʹ) was paired with nested virus-specific reverse primers. PCR for 3ʹ RACE was also done using the Tag1 primer paired with virus-specific forward primers, and nested PCR was performed if needed. All PCR products were sent to Macrogen (South Korea) for Sanger sequencing.

RNA1 and RNA2 of virus isolate 5854 are 5,910 and 4,435 nt long, respectively, excluding the poly(A) tails, and these sequences have been deposited in the GenBank database under the accession codes OR439368 and OR439369, respectively. RNA1 has a single ORF1 of 5,676 nt, which is predicted to encode a 215.0-kDa polyprotein containing 1,892 amino acid (aa) residues. RNA2 has two ORFs in the same translational reading frame (nt 204–3488 and nt 3579–4262), with the first (ORF2A) predicted to encode a 122.2-kDa protein containing 1,095 aa residues, and the second (ORF2B) predicted to encode a 26.3-kDa protein containing 228 aa residues. The presence of the ORF2A stop codon at nt 3486–3488 and the ORF2B start codon at nt 3579–3581 was confirmed by RT-PCR using the primer pair RNA2-3248F/RNA2-4248R (Supplementary Table S1), followed by Sanger sequencing of the amplicon. The 3′-terminal sequence of RNA2 containing the ORF2A stop codon and ORF2B was also verified by 3ʹ RACE. The putative ORF2B protein did not have homology to any other protein in the NCBI protein database based on a BLASTp search, and this ORF is not a conserved feature of members of the genus *Sadwavirus*. It is therefore questionable whether this ORF is translated. Only members of the closely related genus *Torradovirus* have two ORFs in RNA2, but in that genus, the second ORF overlaps the first in a different translational reading frame [5].

Proteinase cleavage sites within each polyprotein were predicted using MUSCLE alignments of sadwaviral polyprotein sequences available in the NCBI protein database (Supplementary Fig. S1). The RNA1 polyprotein contains four putative 3C-like proteinase cleavage sites, H/Y, E/G, Q/S, and Q/G, which lie between the protease cofactor and helicase, the helicase and VPg, the VPg and protease, and the protease and RNA-dependent RNA polymerase (RdRp) proteins, respectively (Fig. 1c). The RNA2 polyprotein is predicted to be cleaved at a T/G dipeptide to give rise to a movement protein and coat protein (Fig. 1c). The cleavage sites E/G, Q/S, and Q/G have been empirically confirmed for strawberry mottle virus and predicted to occur in chocolate lily virus A and dioscorea mosaic associated virus [5, 10], while the cleavage sites H/Y and T/G would be novel.

The 5ʹ untranslated regions (UTRs) of virus isolate 5854 are 71 and 203 nt long for RNA1 and RNA2, respectively, and share 88.7% nt sequence identity. The 3ʹ UTRs are 163 and 173 nt long for RNA1 and RNA2, respectively, and share 87.7% nt sequence identity. Strong sequence similarity between the untranslated regions of RNA1 and RNA2 is a characteristic feature of members of the family *Secoviridae* [5, 9].

To determine the phylogenetic relationships of virus isolate 5854 to other members of the family *Secoviridae*, conserved Pro–Pol protein sequences (from the CG motif of the protease to the GDD motif of the RdRp) were aligned using MUSCLE, and a maximum-likelihood (ML) tree was generated using the RAxML Geneious Plugin version 8, selecting the GAMMA JTT model of protein evolution and a random tree topology to begin the search. Placement of virus isolate 5854 within the subgenus *Cholivirus* of the genus *Sadwavirus* and as a sister taxon to pineapple secovirus A (PSV-A) was supported by very high bootstrap values for the branch nodes (Fig. 2). The current species demarcation criteria for members of the family *Secoviridae* are less than 80% aa sequence identity in the Pro–Pol region or less than 75% aa sequence identity in the coat protein(s) [5]. Isolate 5854 has 64% and 52.9% aa sequence identity in the Pro-Pol and coat protein regions, respectively, to its closest relative, PSV-A, and thus is clearly divergent enough to be classified as a member of a new species in the genus *Sadwavirus*. The name “cattleya purple ringspot virus” (CaPRV) is proposed for the virus, and “*Sadwavirus cattleyacola*” for the formal species name.

**Fig. 2:**
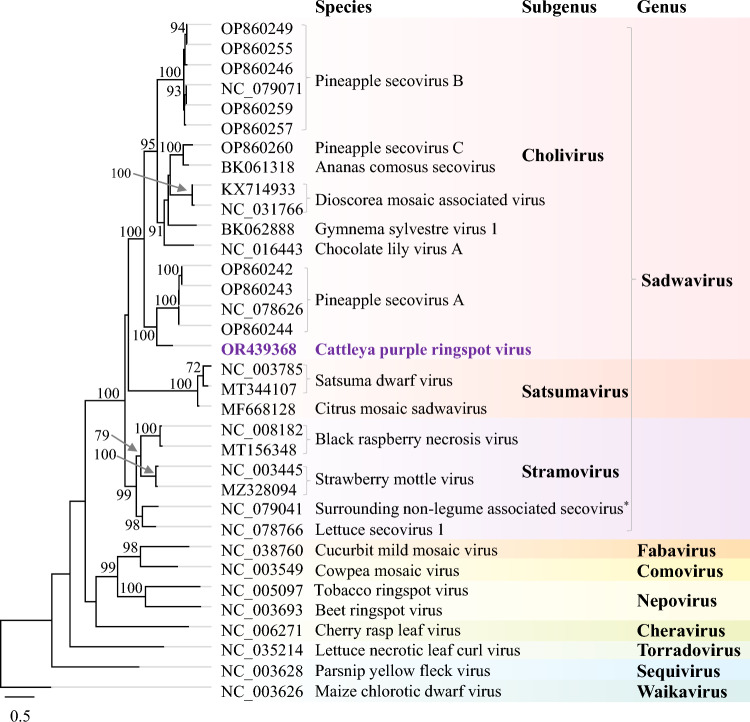
Maximum-likelihood phylogenetic tree showing the relationships between cattleya purple ringspot virus isolate 5854 (in purple font) and members of the genus *Sadwavirus* based on an amino acid sequence alignment of the Pro-Pol region. Tip labels include the GenBank accession number or the RefSeq number, followed by the virus name. Bootstrap values (1000 replicates) greater than 70% are shown at the nodes of the branches. Selected sequences from members of other genera in the family *Secoviridae* were used as outgroups. An asterisk (*) denotes an unclassified member of the family *Secoviridae* (NCBI:txid2734846). The scale bar represents the number of amino acid substitutions per position in the alignment

Further research is required to determine the geographic distribution and host range of CaPRV in Australia and to elucidate its epidemiology. No vector is known for any member of the genus *Sadwavirus*, so it is an intriguing question how these cattleya plants could have become infected. It is conceivable that one or both parental plants could have been asymptomatically infected with the virus and that the virus was seed-transmitted to the progeny plants. Sadwaviruses are also mechanically transmissible, so this could also have provided a route of infection. Additional biological and biochemical characterisation of this virus isolate is needed, particularly to confirm the polyprotein cleavage sites and translation of ORF2B. Furthermore, since the sequence output using the MiSeq platform was relatively low, it is possible that other low-titre viruses might have been associated with the disease symptoms, and high-throughput sequencing on a deeper scale might resolve this possibility.

### Supplementary Information

Below is the link to the electronic supplementary material.

Supplementary file1 (PPTX 51 KB)

Supplementary file2 (DOCX 27 KB)

## Data Availability

The sequence data that support the findings of this study have been deposited in the GenBank database with accession numbers provided in the article.
