# Supplementary material for: Molecular characterisation of a novel sadwavirus infecting cattleya orchids in Australia
Source: Arch Virol. 2024 Mar 7;169(3):68. doi: 10.1007/s00705-024-05980-1 (PMC10920413; doi:10.1007/s00705-024-05980-1)
Supplement: Supplementary file 1 — Supplementary file1 (PPTX 51 KB) [file 705_2024_5980_MOESM1_ESM.pptx]

## Slide 1
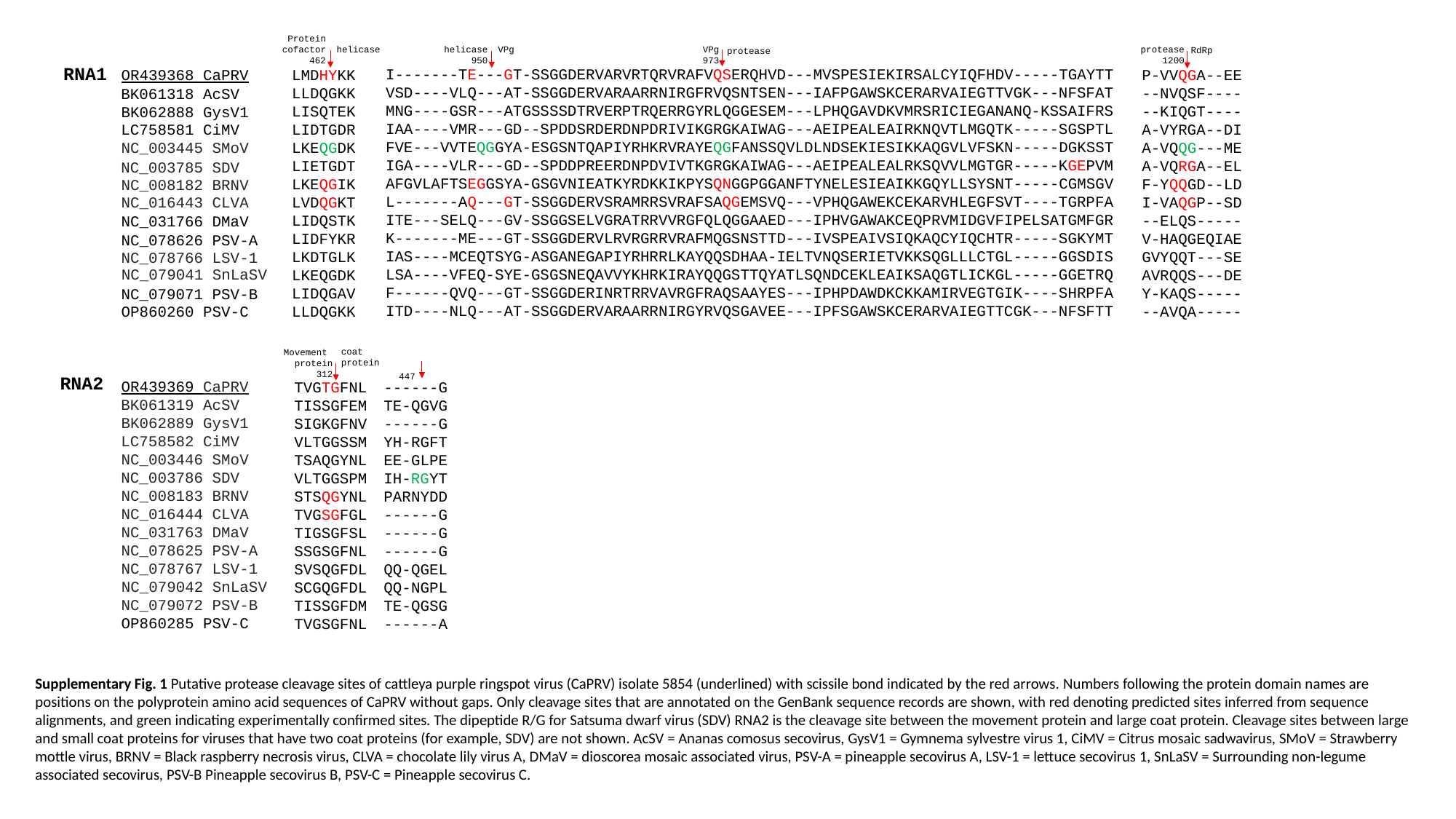

Protein
 cofactor
462
helicase
950
VPg
VPg
973
protease
1200
helicase
RdRp
protease
I-------TE---GT-SSGGDERVARVRTQRVRAFVQSERQHVD---MVSPESIEKIRSALCYIQFHDV-----TGAYTT
VSD----VLQ---AT-SSGGDERVARAARRNIRGFRVQSNTSEN---IAFPGAWSKCERARVAIEGTTVGK---NFSFAT
MNG----GSR---ATGSSSSDTRVERPTRQERRGYRLQGGESEM---LPHQGAVDKVMRSRICIEGANANQ-KSSAIFRS
IAA----VMR---GD--SPDDSRDERDNPDRIVIKGRGKAIWAG---AEIPEALEAIRKNQVTLMGQTK-----SGSPTL
FVE---VVTEQGGYA-ESGSNTQAPIYRHKRVRAYEQGFANSSQVLDLNDSEKIESIKKAQGVLVFSKN-----DGKSST
IGA----VLR---GD--SPDDPREERDNPDVIVTKGRGKAIWAG---AEIPEALEALRKSQVVLMGTGR-----KGEPVM
AFGVLAFTSEGGSYA-GSGVNIEATKYRDKKIKPYSQNGGPGGANFTYNELESIEAIKKGQYLLSYSNT-----CGMSGV
L-------AQ---GT-SSGGDERVSRAMRRSVRAFSAQGEMSVQ---VPHQGAWEKCEKARVHLEGFSVT----TGRPFA
ITE---SELQ---GV-SSGGSELVGRATRRVVRGFQLQGGAAED---IPHVGAWAKCEQPRVMIDGVFIPELSATGMFGR
K-------ME---GT-SSGGDERVLRVRGRRVRAFMQGSNSTTD---IVSPEAIVSIQKAQCYIQCHTR-----SGKYMT
IAS----MCEQTSYG-ASGANEGAPIYRHRRLKAYQQSDHAA-IELTVNQSERIETVKKSQGLLLCTGL-----GGSDIS
LSA----VFEQ-SYE-GSGSNEQAVVYKHRKIRAYQQGSTTQYATLSQNDCEKLEAIKSAQGTLICKGL-----GGETRQ
F------QVQ---GT-SSGGDERINRTRRVAVRGFRAQSAAYES---IPHPDAWDKCKKAMIRVEGTGIK----SHRPFA
ITD----NLQ---AT-SSGGDERVARAARRNIRGYRVQSGAVEE---IPFSGAWSKCERARVAIEGTTCGK---NFSFTT
LMDHYKK
LLDQGKK
LISQTEK
LIDTGDR
LKEQGDK
LIETGDT
LKEQGIK
LVDQGKT
LIDQSTK
LIDFYKR
LKDTGLK
LKEQGDK
LIDQGAV
LLDQGKK
P-VVQGA--EE
--NVQSF----
--KIQGT----
A-VYRGA--DI
A-VQQG---ME
A-VQRGA--EL
F-YQQGD--LD
I-VAQGP--SD
--ELQS-----
V-HAQGEQIAE
GVYQQT---SE
AVRQQS---DE
Y-KAQS-----
--AVQA-----
OR439368 CaPRV
BK061318 AcSV
BK062888 GysV1
LC758581 CiMV
NC_003445 SMoV
NC_003785 SDV
NC_008182 BRNV
NC_016443 CLVA
NC_031766 DMaV
NC_078626 PSV-A
NC_078766 LSV-1
NC_079041 SnLaSV
NC_079071 PSV-B
OP860260 PSV-C
RNA1
coat protein
Movement
protein
312
TVGTGFNL
TISSGFEM
SIGKGFNV
VLTGGSSM
TSAQGYNL
VLTGGSPM
STSQGYNL
TVGSGFGL
TIGSGFSL
SSGSGFNL
SVSQGFDL
SCGQGFDL
TISSGFDM
TVGSGFNL
OR439369 CaPRV
BK061319 AcSV
BK062889 GysV1
LC758582 CiMV
NC_003446 SMoV
NC_003786 SDV
NC_008183 BRNV
NC_016444 CLVA
NC_031763 DMaV
NC_078625 PSV-A
NC_078767 LSV-1
NC_079042 SnLaSV
NC_079072 PSV-B
OP860285 PSV-C
447
------G
TE-QGVG
------G
YH-RGFT
EE-GLPE
IH-RGYT
PARNYDD
------G
------G
------G
QQ-QGEL
QQ-NGPL
TE-QGSG
------A
RNA2
Supplementary Fig. 1 Putative protease cleavage sites of cattleya purple ringspot virus (CaPRV) isolate 5854 (underlined) with scissile bond indicated by the red arrows. Numbers following the protein domain names are positions on the polyprotein amino acid sequences of CaPRV without gaps. Only cleavage sites that are annotated on the GenBank sequence records are shown, with red denoting predicted sites inferred from sequence alignments, and green indicating experimentally confirmed sites. The dipeptide R/G for Satsuma dwarf virus (SDV) RNA2 is the cleavage site between the movement protein and large coat protein. Cleavage sites between large and small coat proteins for viruses that have two coat proteins (for example, SDV) are not shown. AcSV = Ananas comosus secovirus, GysV1 = Gymnema sylvestre virus 1, CiMV = Citrus mosaic sadwavirus, SMoV = Strawberry mottle virus, BRNV = Black raspberry necrosis virus, CLVA = chocolate lily virus A, DMaV = dioscorea mosaic associated virus, PSV-A = pineapple secovirus A, LSV-1 = lettuce secovirus 1, SnLaSV = Surrounding non-legume associated secovirus, PSV-B Pineapple secovirus B, PSV-C = Pineapple secovirus C.
