# Supplementary material for: Molecular characterisation of a novel sadwavirus infecting cattleya orchids in Australia
Source: Arch Virol. 2024 Mar 7;169(3):68. doi: 10.1007/s00705-024-05980-1 (PMC10920413; doi:10.1007/s00705-024-05980-1)
Supplement: Supplementary file 2 — Supplementary file2 (DOCX 27 KB) [file 705_2024_5980_MOESM2_ESM.docx]

**Supplementary Table S1** Virus specific primers used for 5ʹ/3ʹ Rapid Amplification of cDNA Ends (RACE) and confirming ORF2B of cattleya purple ringspot virus (CaPRV) isolate 5854.

| **Experiment** | **Primer name** | **Sequence (5ʹ to 3ʹ)** | **Usage** |
| --- | --- | --- | --- |
| **5ʹ RACE** |  |  |  |
| RNA1 | RNA1-516R1 | CACAGTCGAAATCACGTCAAG | PCR and Sanger sequencing |
|  | RNA1-375R2 | CAGAGTCATGAATAGCTCTATC | Nested PCR and Sanger sequencing |
|  | RNA1-192R3 | GCTCGAGCATATACCTCAAAATC | Nested Sanger sequencing |
| RNA2 | RNA2-833R1 | CAAGTTCATCATTGTGCACATTC | PCR and Sanger sequencing |
|  | RNA2-443R2 | GATAGTGGATTTCGCGAACC | Nested Sanger sequencing |
|  | RNA2-262R3 | TCCACTCGATCTTATTTAACC | Nested Sanger sequencing |
| **3ʹ RACE** |  |  |  |
| RNA1 | RNA1-5362F1 | AGCTCGACGGCAACAGATCT | PCR and Sanger sequencing |
|  | RNA1-5501F2 | GGAATTTGTGAATGATCTCA | Nested PCR and Sanger sequencing |
|  | RNA1-5643F3 | GCTGTGCTTGTGGAAAGAATCAA | Nested Sanger sequencing |
| RNA2 | RNA2-3812F1 | TGTTCCTGCTGGATTTCAATGTG | PCR and Sanger sequencing |
|  | RNA2-3907F2 | GCATGGATTGTTTCGAGATTAA | Nested PCR and Sanger sequencing |
|  | RNA2-4114F3 | TTGCTGATGGAAGAATTTGCA | Nested Sanger sequencing |
| **Confirming ORF2B** | RNA2-3248F | CACGAGGATGCGAACATCTG | RT-PCR and Sanger sequencing |
|  | RNA2-4248R | CACAAAACCCTAATCATCATAACC | RT-PCR and Sanger sequencing |
|  | RNA2-4114R | TGCAAATTCTTCCATCAGCAA | Nested Sanger sequencing |
